# Vacunación infantil: dudas, ambigüedades y toma de decisiones en mujeres-madres de sectores medios de Argentina

**DOI:** 10.1590/0102-311XES010424

**Published:** 2025-02-07

**Authors:** María Jimena Mantilla, Juan Pedro Alonso

**Affiliations:** 1 Consejo Nacional de Investigaciones Científicas y Técnicas, Buenos Aires, Argentina.; 2 Instituto de Investigaciones Gino Germani, Buenos Aires, Argentina.

**Keywords:** Vacunación, Vacilación a la Vacunación, Investigación Cualitativa, Vaccination, Vaccination Hesitancy, Qualitative Research, Vacinação, Hesitação Vacinal, Pesquisa Qualitativa

## Abstract

En los últimos años, las actitudes de rechazo o reticencia a la vacunación y el crecimiento de los llamados “movimientos anti-vacunas” han sido definidos como un desafío para la Salud Pública. El objetivo del artículo es analizar los procesos de decisión de mujeres madres de sectores medios del área metropolitana de Buenos Aires, Argentina, respecto de la vacunación de sus hijos/as, dando cuenta de los factores que disparan actitudes de reticencias o temores a la vacunación. Se realizaron 35 entrevistas en profundidad con mujeres madres de sectores medios de la Ciudad de Buenos Aires, identificadas con estilos de maternidades “naturales” o afines a la denominada “crianza respetuosa”. Se describen diferentes trayectorias o recorridos hacia la reticencia o el rechazo a la vacunación: (a) generadas ya por comentarios o indicaciones de profesionales de la salud durante el parto o controles pediátricos; (b) derivadas de cambios más generales en los estilos de vida, marcados por la opción por cuidados de la salud alternativos o “naturales”; (c) inscriptas en procesos de crítica y desconfianza sobre la biomedicina y la afirmación de la autonomía sobre las decisiones en salud; y (d) a partir de una búsqueda activa de información en fuentes principalmente contrarias a la vacunación. Las dudas o ambigüedades en torno a la vacunación que presentan estas madres no se traducen unívocamente en el rechazo a la vacunación, sino en una diversidad de prácticas: de la vacunación con reparos, la selección o el retraso de algunas vacunas, y la no vacunación.

## Abstract (en)

In recent years, behaviors of rejection or hesitancy towards vaccination and the growth of the so-called “anti-vax movement” have been defined as a challenge to Public Health. This articles aims to analyze the decision-making processes of middle-class mothers in the metropolitan area of Buenos Aires, Argentina, in relation to the vaccination of their children, considering factors that trigger behaviors of hesitancy or fear towards vaccination. A total of 35 in-depth interviews were conducted with middle-class mothers in the city of Buenos Aires, identified with “natural” or similar to the so-called “respectful upbringing” motherhood styles. Different trajectories or paths for vaccine hesitancy or rejection can be traced: (a) previously generated by comments or indications from health professionals during childbirth or pediatric consultations; (b) resulting from more general changes in lifestyles, marked by the option for alternative or “natural” healthcare; (c) inserted in processes of criticism and distrust in relation to biomedicine and the affirmation of autonomy in healthcare decisions; and (d) from an active search for information in sources mostly opposed to vaccination. The doubts or ambiguities regarding vaccination presented by these mothers do not unequivocally translate into the rejection of vaccination, but rather into a diversity of practices: vaccination with reservations, selection or postponement of some vaccines and non-vaccination.

## Resumo

Nos últimos anos, as atitudes de rejeição ou hesitação à vacinação e o crescimento dos chamados “movimentos antivacina” vêm sendo definidos como um desafio para a Saúde Coletiva. O objetivo do artigo é analisar os processos de decisão de mães pertencentes a classe média da região metropolitana de Buenos Aires, Argentina, em relação à vacinação de seus filhos/as, levando em consideração os fatores que desencadeiam atitudes de relutância ou temor à vacinação. Foram realizadas 35 entrevistas em profundidade com mães da classe média da cidade de Buenos Aires, identificadas com estilos de maternidade “naturais” ou afins à denominada “criação respeitosa”. São descritas diferentes trajetórias ou caminhos para a relutância ou rejeição à vacina: (a) geradas previamente por comentários ou indicações de profissionais de saúde durante o parto ou consultas pediátricas; (b) decorrentes de mudanças mais gerais nos estilos de vida, marcadas pela opção por cuidados de saúde alternativos ou “naturais”; (c) inseridas em processos de crítica e desconfiança em relação à biomedicina e de afirmação da autonomia nas decisões de saúde; e (d) a partir de uma busca ativa de informações em fontes majoritariamente contrárias à vacinação. As dúvidas ou ambiguidades relativas à vacinação que estas mães apresentam não se traduzem inequivocamente na rejeição da vacinação, mas sim numa diversidade de práticas: vacinação com restrições, seleção ou adiamento de algumas vacinas e a não vacinação.

## Introducción

En los últimos años, las actitudes de rechazo o reticencia a la vacunación y el crecimiento de los llamados “movimientos anti-vacunas” han sido definidos como un desafío para la Salud Pública a escala global por parte de autoridades sanitarias y organismos internacionales ^1^ ^,^ ^2^ ^,^ ^3^ ^,^ ^4^.

En 2014, el Grupo Asesor Estratégico de Expertos en Inmunización de la Organización Mundial de la Salud (OMS) definió la “reticencia a la vacunación” como la demora en aceptar la vacunación o el rechazo de las vacunas, a pesar de que los servicios de vacunación se encuentren disponibles ^5^. Este concepto plantea un continuo entre quienes confían plenamente en las vacunas y quienes las rechazan de plano, e identifica como “reticentes” a individuos o grupos entre estos polos. Más recientemente, un grupo de trabajo de la OMS definió la reticencia a la vacunación como un estado motivacional de ambivalencia o rechazo a la vacunación ^6^. El concepto de “reticencia” plantea así diferencias con la caracterización de los grupos “anti-vacunas”, término comúnmente utilizado para caracterizar a grupos activistas dedicados a difundir información negativa sobre las vacunas, principalmente a través de internet y redes sociales ^7^ ^,^ ^8^.

Pese al amplio uso del concepto de “reticencia a la vacunación” en políticas públicas e investigación, se han señalado inconsistencias en su definición y operacionalización ^9^ ^,^ ^10^, y cuestionado la validez de utilizar marcos conceptuales diseñados desde países centrales sin considerar seriamente diferencias regionales en las creencias y comportamientos respecto a las vacunas ^11^. A pesar de que resulta complejo cuantificar el problema, la literatura especializada sugiere que, previo a la pandemia por COVID-19, la confianza en las vacunas decrecía a nivel mundial ^12^ ^,^ ^13^. Investigaciones acerca de la reticencia de la vacunación muestran cómo las personas pueden rechazar algunas vacunas y acceder a la aplicación de otras, postergar la vacunación o aceptar las vacunas pero albergando temores ^14^ ^,^ ^15^. Varios estudios muestran que, en el caso de la inmunización infantil, muchos padres y madres que deciden vacunar a sus hijos/as tienen dudas ^5^, y que pueden ser permeables a discursos de grupos que rechazan la vacunación ^14^ ^,^ ^15^.

En Argentina, las vacunas incluidas en el calendario de vacunación son gratuitas y obligatorias ^16^. El esquema es establecido por el Ministerio de Salud, y cuenta con 12 vacunas destinadas niños/as menores de 6 años. Si bien el país ha mostrado altas tasas de vacunación infantil, las coberturas de vacunación mostraban un notorio descenso previo a la pandemia por COVID-19 (una reducción de 10 puntos entre los años 2009 y 2019) ^17^ y, como sucedió en diferentes países, las mismas tuvieron una drástica caída por la pandemia y las medidas aislamiento social: para el año 2020 ninguna de las vacunas del calendario de vacunación infantil superó el 80 % de cobertura ^18^. Asimismo, de acuerdo a varios relevamientos, la confianza y la percepción sobre la seguridad de las vacunas viene disminuyendo en los últimos años ^1^ ^,^ ^13^ ^,^ ^19^ ^,^ ^20^.

Las caídas en la cobertura pueden obedecer a diferentes factores, como problemas de acceso a los servicios de vacunación y/o la falta de insumos, pero en el contexto global de una disminución de la confianza resulta relevante explorar en los factores que inciden en las decisiones de vacunación de los/as niños/as y, más puntualmente, en los procesos involucrados en el desarrollo de actitudes reticentes o de rechazo a la vacunación. Estas actitudes están más extendidas en algunos grupos, como mujeres con mayor nivel educativo, personas identificadas con estilos de vida “alternativos” o “naturales”, entre otros ^21^ ^,^ ^22^ ^,^ ^23^.

Explorar en las perspectivas de estos grupos puede brindar información valiosa sobre los procesos a través de los cuales se gestan las actitudes de dudas o desconfianza hacia las vacunas. Si bien la reticencia a la vacunación viene siendo extensamente estudiada, las investigaciones que exploran en los procesos de toma de decisiones respecto de la vacunación infantil son escasas ^23^ ^,^ ^24^ ^,^ ^25^, en especial en países de América Latina ^26^ ^,^ ^27^.

El objetivo de este artículo es analizar los procesos de decisión de mujeres madres de sectores medios respecto de la vacunación de sus hijos/as, dando cuenta de los factores que disparan actitudes de reticencias o temores a la vacunación de los/as niños/as y la problematización -en el sentido de puesta en cuestión- de las vacunas, y cómo estos procesos inciden en las decisiones de vacunar o no, o de hacerlo en formas alternativas a las indicadas por las autoridades sanitarias.

## Metodología

El estudio siguió un diseño metodológico cualitativo, basado en entrevistas en profundidad con mujeres madres de sectores medios de la Ciudad Autónoma de Buenos Aires y el Gran Buenos Aires. Las entrevistas se realizaron durante el año 2018 y 2019, previo a la pandemia por COVID-19.

Participaron del estudio mujeres identificadas con estilos de maternidades “naturales” o afines a la denominada “crianza respetuosa” o “fisiológica” ^28^ ^,^ ^29^. Las prácticas de vacunación forman parte del repertorio de decisiones que se problematizan en estos grupos, junto a otros procesos como el parto y la crianza, en base a una resignificación de la noción de la “naturaleza” y críticas de la medicalización de estos procesos ^21^ ^,^ ^29^. Se incluyeron mujeres madres de al menos un/a niño/a menor de 6 años y pertenecientes a sectores medios, definidos por una combinación de criterios socioeconómicos y culturales, como un alto nivel educativo, ingreso intermedios y ocupaciones que requieren formación técnica o profesional.

En las entrevistas se exploraron temáticas diversas, como experiencias relacionadas con el parto, la crianza, la atención de la salud y prácticas y creencias respecto a la vacunación infantil. Respecto a la vacunación, se exploraron los procesos a través de los cuales las mujeres llegaron a las decisiones de vacunación (o no vacunación) de sus hijos/as. El artículo focaliza en este último punto.

Las entrevistadas fueron identificadas y contactadas utilizando diferentes vías, como redes sociales ligadas a la temática de la crianza, contactos personales y la técnica “bola de nieve”. Las entrevistas fueron realizadas por la primera autora, tanto en forma presencial como en forma virtual, a través de plataformas como Google Meet (https://meet.google.com).

Las entrevistas fueron grabadas en formato audio y desgrabadas en forma literal. Se realizó un análisis temático de contenido ^30^ con el apoyo del programa ATLAS.TI (http://atlasti.com/). Las entrevistas fueron codificadas siguiendo un abordaje inductivo, para lo cual se desarrolló un manual de códigos. En virtud de los patrones encontrados se generó una tipología de trayectorias y experiencias hacia actitudes reticentes o de rechazo a la vacunación.

El proyecto fue evaluado por el Comité de Ética del Instituto Gino Germani (Resolución 25,319). Previo a la realización de las entrevistas se obtuvo el consentimiento verbal de las participantes.

## Resultados

Participaron 35 mujeres madres de niños/as en edad de vacunación, con una edad promedio de 39 años y un promedio de dos hijos/as (Tabla 1). Las mujeres tenían estudios terciarios o universitarios, y la mayoría se encontraba ocupada ya en forma parcial o a tiempo completo. Varias entrevistadas habían orientado sus carreras profesionales hacia el ámbito del emprendedurismo en el área de servicios de apoyo a la crianza, el embarazo y el parto (como doulas, asesoras, etc.).

**Tabla 1:** Características demográficas de las participantes (N = 35).

Características	n
Edad (años)	
Menores de 35	12
36-40	13
40 o más	10
Nivel educativo	
Terciario completo	18
Universitario completo	17
Número de hijos	
1	14
2	13
3	8

A continuación, se describen diferentes recorridos o trayectorias a través de las cuales las mujeres entrevistadas llegan a dudar de las vacunas, dudas que no necesariamente derivan en la no vacunación. Estos recorridos están marcados por sugerencias de profesionales de la salud, cambios en los estilos de vida y la adopción de cuidados de salud alternativos, procesos de empoderamiento y de crítica a la biomedicina y por la búsqueda activa de información.

### Reticencia a partir de información provista por profesionales de la salud

Una de las primeras vías hacia actitudes de reticencia o rechazo a la vacunación es a través de información o comentarios de profesionales de la salud del ámbito privado, generalmente con orientaciones alternativas (como homeópatas o antroposóficos), realizados en diferentes situaciones y contextos, en los que se pone en duda la necesidad y la seguridad de las vacunas. Estas situaciones ocurren a menudo durante controles en el embarazo. Varias entrevistadas comentaron que diferentes profesionales compartieron información o realizaron comentarios que o bien relativizaban la importancia o la obligatoriedad de la vacunación y su eficacia como sistema de protección inmunológica. En otros casos, las dudas y temores surgen luego del parto, cuando en la consulta con pediatras estos sugieren la asociación entre la vacunación y alguna condición o problema de salud, o comparten contenidos de grupos que rechazan las vacunas. 

Una de las informantes mencionó que las dudas iniciales respecto a la vacunación de su hija comenzaron cuando la partera, en una consulta previa al parto, les advirtió que “*si eligen no vacunar no los dejen desprotegidos, utilicen homeopatía o algún tipo de medicina natural*”. Esta revelación, si bien no la llevó a no vacunar a su beba, generó una duda que se profundizó aún más durante una consulta postnatal con una pediatra homeópata que le aseguró que la causa de los cólicos de la beba era una intoxicación por las vacunas recibidas durante el embarazo.

En otros casos, las dudas surgen durante las consultas pediátricas. Otra de las entrevistadas comenzó a dudar sobre la conveniencia de la vacunación cuando llevó a su primer hijo de dos años a una pediatra antroposófica preocupada por su tardanza en caminar, quien asoció esta situación a los efectos de las vacunas. Esta información sembró la primera inquietud que la llevó a decidir no vacunar a su segunda hija. En sus propias palabras, y en relación a su primer hijo: “*vacunarlo fue la cosa que más me arrepiento desde que fui madre*”.

En estos casos el camino hacia la desconfianza a las vacunas y la preocupación por los supuestos daños de la vacunación es inspirada por profesionales de la salud, muchas veces consultados por temáticas inclusive ajenas a la vacunación.

### Reticencia como continuidad con cambios en estilos de vida

Una segunda vía a través de las que se llega a problematizar o cuestionar la vacunación está ligada a cambios más generales en el estilo de vida en los que se enmarcan las decisiones respecto a la propia salud y la salud de los hijos.

En algunos de estos casos, estas elecciones previas contribuyen a que se generen dudas y temores sobre la vacunación que, si bien no alcanzan para decidir no vacunar, llevan a que se posponga o se dilate la decisión de vacunación, y con ello a incumplir los calendarios y recomendaciones. Es el caso de Florencia, quien vacunó a su primera hija sin mayores cuestionamientos, pero durante el embarazo de su segundo hijo ya había ingresado al universo del parto respetado y comenzado a cuestionar determinadas prácticas médicas. De esta manera narra su experiencia:

“*Yo venía de la cultura de que vacunar es lo que hay que hacer. Pero con mi segundo hijo más que nada me daba como temor, no me animaba a vacunarlo, fue todo el tiempo: ‘bueno, tengo que ir’. Quería vacunarlo después de los seis meses, pero a partir de los seis meses en adelante es como que me costaba un montón. Por el hecho de que traiga alguna consecuencia. Después también un poco es: ‘bueno, prefiero que al principio esté con teta y desarrolle el sistema inmunológico y nada... pero siempre dudando’*” (Entrevista a Florencia).

Cuando su hijo cumplió dos años y comenzó su escolarización, finalmente decidió vacunarlo, motivada por el temor al contagio de enfermedades. Esta ambigüedad: el miedo a vacunar y también a no hacerlo, es parte constitutiva de la experiencia emocional que acompañan a varias entrevistadas.

En algunos casos, sin embargo, el cuestionamiento a la vacunación, y la decisión de no vacunar, parecieran derivarse de procesos cuasi irreflexivos, sin peso de ambivalencia añadido, que se toman como parte de una elección de estilo de vida. Marta, por ejemplo, recién con su tercera hija, optó por la no vacunación. Fue en ese embarazo que se formó como doula y en ese contexto empezó a problematizar la vacunación. Así describe el proceso de llegada a la decisión:

“*Pero fue como que se fue dando, no fue demasiado la pregunta de vacunarnos o no. Bueno, ‘no, ahora no vacunamos’ y ya se pasaron, no sé, seis meses, ¿y para qué vamos a hacerlo? La verdad que también han ingresado a este colegio* [de orientación alternativa] *que tampoco era como una condición estar vacunado y fue quedando. Si en algún momento ella cree que se tiene que vacunar, se vacunará, no sé, lo decidirá ella cuando sea mayor si piensa que tiene que vacunarse*” (Entrevista a Marta).

La expresión “se fue dando” grafica este modo de llegar a la decisión de no vacunar o postergar la vacunación sin que esta se inscriba en un espacio racional, de evaluación de pros y contras, o siquiera de búsqueda de información. De hecho, el argumento acerca de “esperar a que ella sea mayor” revela la falta de información respecto de las razones sanitarias e inmunológicas en las que se basan las edades y calendarios de vacunación. En este tipo de casos, la semilla de la problematización está planteada, de antemano, en el propio proceso de ingresar a un mundo de prácticas (alimentarias, de cuidado de la salud y el embarazo, de crianza) que se alinean con la no vacunación. La circulación en contextos y círculos que avalan estas decisiones, como la consulta a pediatras que desaconsejan la vacunación obligatoria y una elección educativa que facilita también esta decisión al no exigir certificación de vacunas para el ingreso escolar, contribuyen a que estas prácticas no se vean confrontadas.

### Dudas sobre la vacunación basadas en procesos de empoderamiento y críticas a la biomedicina

En otros casos, las dudas y cuestionamientos a la vacunación se inscriben en procesos previos de críticas a los sistemas de atención biomédica y de reconocimiento de las capacidades corporales y de valoración de la autonomía en las decisiones en salud. Son mujeres que, a partir de la gestación y/o después del parto, comenzaron un proceso de autoconocimiento corporal y búsqueda de gestionar por sí mismas las decisiones de salud. Este proceso de empoderamiento emerge en algunas trayectorias como producto de una vivencia traumática en el parto o por la opción por partos no institucionalizados. La experiencia de una de las entrevistadas ilustra estos procesos de crítica a la biomedicina relacionados a la atención del parto sobre los que luego se apoya la puesta en cuestión de las vacunas:

“*Bueno, la verdad es que a mí me empoderó un montón la cesárea porque en algún lugar me llené de impotencia. Pero esa impotencia me dio fuerza como para decir: ‘bueno, esto no pude, pero a partir de ahora yo voy a decir las cosas claramente, varias veces, no voy a permitir que le hagan intervenciones a mi hija’. Entonces ya desde el principio no permití que le pongan las vacunas en la institución, les dije que no, que las iba a poner más tarde*” (Entrevista a Mariana).

Como en otros casos, una cesárea considerada innecesaria, o la percepción de haber sufrido violencia obstétrica durante el parto, marca una bisagra y produce una suerte de revisión que lleva a las mujeres a cuestionar los procesos de atención de la salud de sus hijas/os. Algunas mujeres comienzan a percibir algunas prácticas médicas como “violentas” o “invasivas”. En particular, varias mujeres que optan por un parto domiciliario, describan la negativa “al pinchazo” de los recién nacidos como el inicio de una actitud de rechazo de la vacunación. La experiencia de Verónica ilustra este tipo de recorridos:

“*Porque empecé a informarme de las vacunas a través del primer parto en casa y no quería la invasión del pinchazo, como que me parecía terrible, empecé a decir: ‘claro, por qué se vacuna a un bebé así, ¿no?, con esa urgencia, ¿qué pasaría si la puedo vacunar después, no en el momento del parto?’ Entonces esa fue como la primera información, que, si iba a decidir vacunarlas, las podía vacunar después. Y por ahí ese después empezó a transcurrir en el puerperio y fueron como pasando los días*” (Entrevista a Verónica).

Para las mujeres que buscaron experiencias de parto sin intervenciones médicas innecesarias (como cesáreas, episiotomías de rutina, etc.), el hecho de vacunar al recién nacido es interpretado como otra de las prácticas médicas violentas que asocian a los partos institucionales (en ocasiones, a partir de experiencias de maltrato), previamente problematizadas por estas mujeres. Este tipo de experiencias, común a varias informantes, marca el inicio de un cuestionamiento progresivo (de carácter más vivencial que fundamentado en razones esgrimidas racionalmente) que tiene como resultado final la decisión de no vacunar o postergar la vacunación en virtud de una mayor confianza en una protección y cuidado de la salud de carácter natural mediado por la lactancia, y las dimensiones vinculares del cuidado. Como menciona Verónica:

“*Pero después, qué sé yo, si le agarraba un moco o algo, me agarraba como cosa, pero no sé, empecé a ser consciente de cuán sano era el vínculo, cuerpo a cuerpo, porque... no sé, por ahí tenía fiebre y la recomendación era: ‘bueno, quedate desnuda y ponétela cuerpo a cuerpo, con el fular hasta que se autorregulen’, y así dejé de usar ibuprofeno. Entonces fue como todo un paso a paso, descubrir otras formas de cuidado*” (Entrevista a Verónica).

### Reticencia a partir de la búsqueda activa de información

Un último tipo de llegada a las dudas o rechazo a la vacunación se produce a partir de una búsqueda activa de información, ya mediante la lectura de artículos científicos, *blogs* o páginas de grupos opuestos a la vacunación, o charlas de referentes de estos grupos o de la comunidad “antivacunas”, que difunde activamente contenidos negativos sobre la vacunación a través de diferentes medios y estrategias. En estos casos, más que una duda o un conocimiento casi intuitivo y vivencial, la decisión de vacunación se inscribe en un proceso más reflexivo, mediado o influido por la información adquirida a partir de estas fuentes. Como menciona una entrevistada:

“*He participado en algunos seminarios, en charlas informativas sobre vacunas que dan distintas personas, y entiendo la base sobre la que se construyó el paradigma de la vacuna y quienes explican por qué no sirven los argumentos que dan y la evidencia que presentan detrás de esto, y creo bastante en eso, de hecho cada vez me convenzo más de la idea de no vacunar, y cada vez más miedo me dan las vacunas, que Argentina tenga un calendario tan extenso en vacunas no me parece casual*” (Entrevista a Alejandra).

En estos casos, la reticencia a la vacunación se basa en la circulación de información sobre sus supuestos efectos secundarios, dudas sobre su efectividad en la prevención de enfermedades, y sobre aspectos de la política de inmunización, como la amplitud del calendario de vacunación o su obligatoriedad. Como también argumenta Alejandra:

“*Que Argentina tenga un calendario tan extenso en vacunas no me parece casual. Me parece que justamente somos el conejillo de Indias del mundo, porque en otros países del primer mundo no te permiten ni ese calendario de vacunas, que acá las dan y en otros lados están prohibidas, y que también la vacuna es una opción, y acá no es una opción, entonces hablamos de mi cuerpo es mío, mi cuerpo mi decisión, y en vacunas le permitimos al Estado obligarnos a introducirnos materiales dentro del cuerpo que pueden ser potencialmente tóxicos*” (Entrevista a Alejandra).

En este sentido, las decisiones para este grupo de mujeres se presentan como decisiones informadas (usando como fuente a grupos que rechazan la vacunación), en los que se ponen en cuestión ya todas las vacunas recomendadas o algunas vacunas, en ocasiones acompañadas por el criterio de profesionales que avalan o recomiendan estas prácticas de vacunación selectiva. Así lo explica Yamila:

“*La pediatra con la que ahora estamos es una pediatra que no es antivacunas, sino que simplemente acompaña decisiones informadas, ella aconseja o desaconseja determinadas vacunas y te dice, ‘yo sé que, en determinados momentos, porque hay epidemia, lo que sea, hay vacunas que sí hay que dar y otras que, yo sabiendo cómo vive tu hija, dónde vive tu hija, hay vacunas que no van a ser necesarias’*” (Entrevista a Yamila).

En suma, en este tipo de recorrido se llega a la decisión a partir de la desconfianza en la toxicidad de las vacunas y las dudas sobre la efectividad como medida preventiva de enfermedades; desconfianza que se genera (y luego profundiza) a partir del consumo de información provista por grupos que rechazan la vacunación, ya a través de redes sociales como páginas web. Por ello, se trata de una decisión que se asume sin mayores dudas, inscripta en una trayectoria previa de problematización del parto y la crianza, a la que se le añade un proceso de investigación activa respecto de los riesgos de las vacunas.

## Discusión

En este trabajo analizamos los procesos de toma de decisiones de mujeres madres de sectores medios del área metropolitana de Buenos Aires sobre la vacunación de sus hijos/as. Exploramos los factores que inciden en estas decisiones, dando cuenta de diferentes trayectorias y recorridos que llevan a la problematización de la vacunación y al surgimiento de dudas y temores. Las trayectorias hacia la reticencia o el rechazo a la vacunación se producen ya por comentarios o indicaciones de profesionales de la salud durante el parto o controles pediátricos; se derivan de cambios más generales en los estilos de vida, marcados por la opción por cuidados de la salud alternativos o “naturales”; se inscriben en procesos de crítica a la biomedicina y la afirmación de la autonomía sobre las decisiones en salud; o se dan a partir de una búsqueda activa de información en fuentes principalmente contrarias a la vacunación.

Estos procesos de puesta en cuestión o problematización de la vacunación se inscriben en un contexto sociocultural donde se conjugan creencias sobre el cuerpo, la salud, la autonomía y la inmunidad. En el artículo se reponen los contextos y el momento en el que emergen estas dudas o temores, los actores y las fuentes que intervienen en estos procesos, que no se traducen unívocamente en el rechazo a la vacunación sino en una diversidad de prácticas: de la vacunación con reparos, la selección o el retraso de algunas vacunas, y la no vacunación.

Como ha sido descripto, el perfil de las madres o padres con reticencias o dudas sobre la vacunación no es un grupo homogéneo ^15^ ^,^ ^26^. Las personas tienen dudas o temores sobre las vacunas por una variedad de motivos, y estas dudas no siempre se correlacionan con el rechazo de la vacunación. Sin embargo, los grupos o colectivos opuestos o reticentes a la vacunación suelen describirse como grupos homogéneos tanto respecto a las prácticas como a las actitudes ^21^ ^,^ ^22^.

El grupo de mujeres que comparten ciertos valores y prácticas ligadas a lo que se define como “maternidades naturales” o “crianza respetuosa”, marcadas por un estilo de vida “alternativo” y una concepción de la salud en la que se valora lo “natural”, suele ser uno de ellos. En su investigación sobre madres que se oponen a la vacunación infantil en Estados Unidos, Reich ^21^ menciona que estas son percibidas como fuentes de toxicidad, desde una perspectiva que opone fuertemente los medios “naturales” de cuidado de aquellos percibidos como “artificiales”. Algunas prácticas de estos grupos, como la lactancia prolongada, el consumo de comida orgánica, etc., son consideradas como medios superiores y “naturales” de fortalecer la inmunidad de sus hijos/as. En estudios sobre las decisiones de no vacunación por madres y padres de São Paulo, Brasil, Barbieri & Couto ^23^ y Barbieri et al. ^31^ también sostienen esta centralidad de los estilos de vida saludables en el rechazo a las vacunas, y la asociación entre este rechazo y prácticas como las opciones por partos no institucionales y ciertos estilos de crianza. En líneas generales, estos grupos de mujeres son caracterizados por el rechazo a la vacunación, basada en decisiones informadas y la búsqueda activa de información, y en miradas holísticas sobre la salud, la enfermedad y la prevención, y la preferencia por tipos de atención “alternativas” ^26^.

En nuestro estudio con mujeres que se identifican a sí mismas en base a estas coordenadas culturales, identificamos que los grados de reticencia y rechazo de las vacunas son variables, al igual que los motivos y los recorridos hacia estas actitudes y prácticas.

Por un lado, la llegada a actitudes reticentes o de rechazo no necesariamente se producen luego de un proceso informado, como muestran varios estudios que describen a estos grupos de mujeres como buscadoras activas de información y a las decisiones de vacunación como decisiones informadas ^21^ ^,^ ^23^ ^,^ ^32^. En nuestro estudio, salvo un grupo de mujeres que se acerca a este perfil, las informantes enfatizan sus vivencias personales y ponen en juego sus creencias en torno a la salud, el cuidado y la autonomía como base de sus reticencias. Para la mayoría de las entrevistadas, la llegada a actitudes de reticencia e incluso de rechazo no se basa en el acceso a información sino a procesos menos reflexivos, como la percepción de la práctica de vacunación como “violenta”, impuesta, o que no está en consonancia con su estilo de vida y el que quieren para sus hijos/as. En algunos casos, incluso el acceso a información difundida por grupos que rechazan las vacunas es posterior a las decisiones de no vacunar o aplazar la vacunación.

El germen de la desconfianza o dudas con las vacunas a menudo se genera durante el embarazo, y en la búsqueda de partos “humanizados” o no institucionales, como también muestran los estudios citados ^21^ ^,^ ^23^. El cuestionamiento a las rutinas en la atención del embarazo y el parto, ya durante estos procesos como luego de malas experiencias, movilizan actitudes críticas a la biomedicina que impactan en la percepción y las actitudes sobre las vacunas.

Tanto en estos casos de desconfianza a la vacunación a partir de actitudes críticas sobre la medicina como de la búsqueda más activa de información, en los que las decisiones de vacunación parecen problematizarse más conscientemente, se alinean con procesos contemporáneos de procesos de mayor participación e información de los/as pacientes y usuarios/as de los servicios de salud. Este rol más activo en el proceso de decisión sobre vacunación, se opone tanto a algunas formas más “pasivas” de aceptar la vacunación, como de algunos recorridos de no vacunación o de postergación de la vacunación en los que no hay una decisión explícita de no vacunar, sino que estas prácticas se desprenden de otras decisiones, como la opción por partos domiciliarios o la atención con profesionales de la salud que no indican las vacunas.

Al respecto, los/as profesionales de la salud con orientación alternativa, en ocasiones desaconsejando en forma abierta la aplicación de todas o algunas vacunas, o avalando decisiones como la postergación de la vacunación, también juegan un rol relevante en las decisiones de no vacunación. La importancia de las recomendaciones de los profesionales de la salud en la toma de decisiones respecto a la vacunación ha sido ampliamente documentada ^24^ ^,^ ^25^ ^,^ ^32^. Al respecto, la atención durante el embarazo se destaca como un momento importante en las decisiones de vacunación de los recién nacidos que, en algunas ocasiones, pueden tener un alcance más amplio, e impactar en vacunas posteriores ^33^. El rechazo a la vacunación de los recién nacidos de algunas madres, por temor al “pinchazo”, o para evitar una práctica considerada como “violenta” al momento de nacer, limita las prácticas de vacunación ya no por un rechazo de la vacunación en sí, sino por su oportunidad, siendo a menudo un motivo para postergarlas o aplazarlas. Estas madres no se consideran “anti-vacunas”, y estos aplazamientos en ocasiones se constituyen en trayectorias de no vacunación, donde el momento de una decisión sobre la vacunación se posterga.

La prevalencia de dudas o temores sobre la vacunación no necesariamente se traduce en el rechazo a la vacunación, tal como sugiere las mismas definiciones de reticencia a la vacunación y diferentes estudios ^1^ ^,^ ^15^ ^,^ ^26^. Si bien varias de las entrevistadas no han vacunados a sus hijos/as, otras lo han hecho pese a esas dudas, o han pospuesto o elegido vacunar solo con algunos biológicos. En la mayoría de los casos, las decisiones sobre la vacunación, excepto para aquellos que se oponen de forma más enfática, están marcadas (en algún momento) por la preocupación tanto por los riesgos asociados a la vacunación como por el temor a no vacunar (o no haber vacunado) y a exponer a sus hijos/as a enfermedades graves que podrían prevenirse. Estas preocupaciones oscilan entre ambos extremos, reflejando las formas contemporáneas en las que, según Furedi ^34^, se manifiesta la sobreestimación de los riesgos experimentados por los padres en la contemporaneidad. En nuestro estudio, las mujeres describen el peso de tomar decisiones para las cuales no siempre tienen las herramientas adecuadas. La contracara de la valoración de la autonomía y la recuperación del poder sobre sus cuerpos y la gestión de la salud se vislumbra en procesos a veces conflictivos o temerosos al enfrentar decisiones relacionadas con la vacunación.

A menudo, el fenómeno de la reticencia a la vacunación se asocia en forma directa con los llamados grupos “anti-vacunas” ^8^. Varios estudios han señalado la necesidad de revisar esta caracterización de algunas ansiedades y preocupaciones respecto a la vacunación, considerando en parte la divergencia al interior de los grupos que cuestionan las vacunas y los procesos sociales más amplios que han derivado en la puesta en cuestión de las vacunas por una parte de la población ^7^ ^,^ ^35^. Asimismo, si bien el acceso a los discursos de grupos que rechazan la vacunación tiene un impacto en la percepción de las vacunas, en nuestro estudio observamos diferencias en la forma en que las entrevistadas se relacionan con estos discursos y su influencia en las decisiones. Si para algunas mujeres el acceso a este tipo de contenidos ha forjado actitudes de rechazo a las vacunas, en otras trayectorias han tenido un peso menor, y a lo sumo respaldan decisiones ya tomadas o alimentan dudas o temores que no necesariamente se traducen en decisiones de no vacunación.

El estudio tiene algunas limitaciones. La selección intencional de un perfil particular de mujeres, asociadas a tipos de crianza natural y a un sector social, no permite generalizar los procesos de toma de decisiones respecto a la vacunación y los factores que inciden en la emergencia de actitudes reticentes o de rechazo a la vacunación. La selección de mujeres-madres obedeció a que, tal como sugiere la literatura, son quienes mayormente implicadas en las decisiones sobre el cuidado de la salud ^19^, pero no necesariamente refleja la actitud de los padres. Asimismo, el estudio aborda percepciones y prácticas sobre la vacunación previas a la pandemia por COVID-19 y la implementación de las vacunas por COVID-19, cuyo impacto en la reticencia a la vacunación y en los procesos de toma de decisión aún debe estudiarse ^36^.

Pese a estas limitaciones, el estudio aporta la descripción de los procesos a través de los cuales emergen dudas y temores en relación a la vacunación infantil, y al entendimiento de la influencia de estos temores en la toma de decisiones. Factores como el rol de los profesionales, las percepciones sobre la biomedicina y la autonomía, los estilos de vida, y la confianza en la seguridad y efectividad de las vacunas se interrelacionan en los recorridos, y decisiones de vacunación, atravesados por dudas y faltas de certezas que no se traducen de forma unívoca a prácticas de vacunación.
